# A Role for FACT in Repopulation of Nucleosomes at Inducible Genes

**DOI:** 10.1371/journal.pone.0084092

**Published:** 2014-01-02

**Authors:** Warren P. Voth, Shinya Takahata, Joy L. Nishikawa, Benjamin M. Metcalfe, Anders M. Näär, David J. Stillman

**Affiliations:** 1 Department of Pathology, University of Utah Health Sciences Center, Salt Lake City, Utah, United States of America; 2 Massachusetts General Hospital Cancer Center, Charlestown, Massachusetts, United States of America; 3 Department of Cell Biology, Harvard Medical School, Boston, Massachusetts, United States of America; Texas A&M University, United States of America

## Abstract

Xenobiotic drugs induce Pleiotropic Drug Resistance (*PDR*) genes via the orthologous Pdr1/Pdr3 transcription activators. We previously identified the Mediator transcription co-activator complex as a key target of Pdr1 orthologs and demonstrated that Pdr1 interacts directly with the Gal11/Med15 subunit of the Mediator complex. Based on an interaction between Pdr1 and the FACT complex, we show that strains with *spt16* or *pob3* mutations are sensitive to xenobiotic drugs and display diminished *PDR* gene induction. Although FACT acts during the activation of some genes by assisting in the nucleosomes eviction at promoters, *PDR* promoters already contain nucleosome-depleted regions (NDRs) before induction. To determine the function of FACT at *PDR* genes, we examined the kinetics of RNA accumulation and changes in nucleosome occupancy following exposure to a xenobiotic drug in wild type and FACT mutant yeast strains. In the presence of normal FACT, *PDR* genes are transcribed within 5 minutes of xenobiotic stimulation and transcription returns to basal levels by 30–40 min. Nucleosomes are constitutively depleted in the promoter regions, are lost from the open reading frames during transcription, and the ORFs are wholly repopulated with nucleosomes as transcription ceases. While FACT mutations cause minor delays in activation of *PDR* genes, much more pronounced and significant defects in nucleosome repopulation in the ORFs are observed in FACT mutants upon transcription termination. FACT therefore has a major role in nucleosome redeposition following cessation of transcription at the *PDR* genes, the opposite of its better-known function in nucleosome disassembly.

## Introduction

Fungal disease remains a serious clinical concern, notably in an increasingly immunocompromised population, and especially given newly emerging fungal pathogens [1]–[3]. Drug treatment using an array of antifungal compounds often becomes ineffectual due to the ability of pathogens to overproduce membrane-associated broad-specificity xenobiotic efflux pumps, rendering them resistant to therapy [4]. These transport proteins are members of the ubiquitous and diverse ATP Binding Cassette (ABC) superfamily, which have a broad spectrum of roles in many phyla, including MDR (Multi-Drug Resistance) in cancer chemotherapy, antibiotic, herbicide, and other cytotoxic compound resistance, as well as in cellular homeostatic processes [5]–[9]. ABC members in *Saccharomyces cerevisiae* are similarly diverse, but the subset that confers resistance to antifungal toxins are encoded by the *PDR* (Pleiotropic Drug Resistance) genes [10]–[14].

Xenobiotic-induced expression of PDR efflux pump genes is complex, but is primarily programmed by the zinc-cluster transcription factor Pdr1 [15]–[17], along with the orthologous Pdr3 factor [18], [19], which are constitutively bound to PDRE sequences in PDR promoters, even in the absence of toxin [20]. Gene regulatory cofactors including the histone modifier SAGA and chromatin remodeler Swi/Snf have been shown to be recruited to PDR target gene promoters, and implicated in their activation [20]. Screens for mutations causing greater *PDR1*-dependent drug sensitivity have also indicated the HDAC Rpd3L may have a role in PDR gene activation [21]. Our previous work demonstrated that Pdr1 is capable of binding directly to antifungals and other xenobiotics, presumably causing a conformational change liberating the carboxyl-terminal transactivation domain, which can then productively engage transcription coactivators [22], [23]. We also showed that xenobiotic-activated Pdr1 promotes recruitment of the Mediator coactivator to the *PDR5* promoter through direct interaction of the KIX domain in its Gal11 component, and that Gal11 and other modules of Mediator are necessary for the PDR response in both *Saccharomyces* as well as pathogenic *Candida* yeasts [22], [23].

FACT (FAcilitates Chromatin Transcription [24]) is an abundant, essential, highly conserved chromatin altering factor in eukaryotes composed of a heterodimer of Spt16 [25] and the HMG domain containing SSRP1 [26], [27] in non-fungal eukaryotes. In fungi, Spt16 complexes with the SSRP1 truncated homolog Pob3 but the HMG domain lies in a separate protein, Nhp6 [28], that associates with the FACT core supra-stoichiometrically [29]–[31]. Both Spt16 and Pob3 each carry a double pleckstrin homology (PH) domain as well as other protein binding domains [32], [33]. FACT is able to reorganize the interaction of DNA with histones in a nucleosomal context in vitro, but in a way mechanistically different than ATP-dependent remodelers [34]. Lack of FACT function in vivo results in a number of both replication and transcription phenotypes [31], [35]–[37]. A role for FACT in transcriptional elongation is supported by its ability to stimulate transcription through a chromatin barrier in vitro [24], its association with elongation factors [38], [39], and the presence of FACT at transcribed regions of genes in vivo [40], [41]. FACT is modeled as both disassembling and reassembling nucleosomes during elongation [42], with the importance of the reassembly role demonstrated by the observation that FACT mutants activate cryptic TATA elements because of inadequate restoration of repressive chromatin following transcription [40], [43]. There is also evidence that FACT regulates transcription initiation by affecting the accessibility of promoter regions to initiation factors and by participating in nucleosome eviction [37], [44]–[47]. FACT has therefore been implicated in both removal and assembly of nucleosomes prior to and during transcription elongation.

In this study, we demonstrate that efficient activation of PDR genes requires the chromatin reorganizing complex FACT, and that yeast defective in FACT function show increased sensitivity to xenobiotic drugs. PDR genes respond to toxin exposure with a rapid accumulation of PDR mRNA quickly followed by a subsequent reduction in transcript levels. This transcriptional induction and reduction is mirrored by sudden histone loss and repopulation across PDR loci, which allows direct analysis of FACT's effects on chromatin assembly and disassembly in a native context. PDR gene promoters contain Nucleosome Depleted Regions (NDRs), and thus FACT is not required for the same types of chromatin changes that occur at other promoters before gene activation, although FACT mutations cause minor delays in activation of *PDR* genes. Surprisingly, FACT mutants instead display a marked delay in repopulation of nucleosomes in the proximal promoter and open reading frame following cessation of transcription, revealing a novel role of FACT in chromatin reassembly in the wake of gene transcription.

## Materials and Methods

For large-scale purification and identification of GST–Pdr1 AD-associated proteins, 6 L YPD medium (with 3% dextrose) was inoculated with yeast at an OD600 of 0.03 and grown at 30°C with shaking for approximately 13 hours, until an OD600 of ∼4. The cell pellet was then resuspended in 0.25 volumes of lysis buffer (50 mM Tris-HCl (pH 8.0), 400 mM NaCl, 5 mM MgCl_2_, 1 mM EGTA, 1 mM EDTA, 0.1% NP40, 1 mM DTT, 0.25 mM PMSF, 1 mM benzamidine, 0.5 mg ml-1 aprotinin and Protease Inhibitor Cocktail (Complete, Roche)), and the suspension was quick-frozen in liquid nitrogen. Frozen cells were lysed by grinding with a mortar and pestle together with dry ice. One volume of lysis buffer was added after evaporation with dry ice, and the extract was spun at 4,000 g for 10 min. The supernatant was pre-incubated with 200 µl glutathione–Sepharose-bound GST for 2 h at 4°C with rotation, then it was incubated for another 3 h at 4°C with 200 µl glutathione–Sepharose-bound GST–Pdr1-AD (amino acids 966–1068), expressed and purified as described [22]. The beads were washed seven times with wash buffer (20 mM Tris-HCl, pH 8.0, 250 mM KCl, 0.1 mM EDTA, 10% glycerol, 0.1% NP-40, 5 mM MgCl_2_, 1 mM EGTA, 1 mM EDTA, 1 mM DTT, 0.25 mM PMSF, 1 mM benzamidine and Protease Inhibitor Cocktail). The beads were finally washed once with low salt (150 mM NaCl) wash buffer. The bound proteins were then eluted with 500 μL of 0.3% sarkosyl in binding buffer for 1 h at 4°C and dialyzed overnight in 1 L of dialysis buffer (1% SDS, 1 mM β-mercaptoethanol and 1 mM Tris-HCl, pH 8.0). The dialyzed eluate was concentrated by dry-ice/ethanol SpeedVac to approximately 80 µl. The eluted proteins were resolved on 10% polyacrylamide gel and stained with Coomassie colloidal blue. Stained proteins were excised and subjected to trypsin digestion, followed by liquid chromatography MS/MS (LC-MS/MS) at the Taplin Biological Mass Spectrometry Facility at Harvard Medical School as previously described (Thakur et al. 2008).

Yeast strains and plasmids are listed in Table S1. For plate spot dilution growth assays, liquid cultures of the indicated strains at permissive temperature were grown to saturation, serially diluted in 10 fold increments, spotted onto YPAD agar media containing 0, 200, or 500 nM Ketoconazole (Sigma), incubated as denoted, and photographed.

Liquid culture growth rates were measured by diluting stock cultures grown for 24 hr at 25°C from single colonies in quadruplicate into fresh YPAD containing 0, 200, or 500 nM Ketoconazole, and incubating all cultures simultaneously in a Bioscreen-C Automated Growth Curve Analysis System (Growth Curves USA, Piscataway, NJ) for 24 hr at the indicated temperatures. Data consisting of OD_600_ measurements at 30 min intervals were analyzed and plotted using Prism 6.0c (GraphPad Software).

For gene expression and ChIP analysis, cells were cultured at the indicated temperature in 750 mL YPAD for 12–20 hr to a density of ∼1×10^7^ cells/mL (OD_660_ = ∼0.75). For analysis of induction of PDR genes, aliquots were collected just prior to, and every 2.5 to 10 min after addition of Ketoconazole (Sigma) to 40 µM. ChIP samples were prepared by immediately adding formaldehyde at the designated time to a 50 mL culture aliquot to 1%, rocking at RT for 20 min, adding glycine to 125 mM, and immediately chilling on ice for 24 hr. Cells were then rinsed by centrifugation and resuspension twice in 1 volume of PBS, once in 1/50 volume of FA buffer [48], and the pelleted cells were flash frozen in liquid nitrogen, and stored at −80°C for processing.

For Myc, histone H3, and Spt16 immunoprecipitations, chromatin was prepared by cell lysis in 750 µL ice-cold ChIP Lysis Buffer (FA buffer containing 0.1% NP-40, 0.1% SDS, 5 mM MgCl_2_, 1 mM DTT, and protease inhibitors), and 1.2 mL pre-chilled 0.5 mm Zirconia/Silica beads in screw-cap microcentrifuge tubes using 6 cycles of 2 min each in a Mini-Bead-Beater 96 (Biospec Products), in a block chilled to −20°C, with 1 min chilling of the tubes on ice between each cycle. Recovered lysates were centrifuged 1 hr at 23 K rpm at 0°C, supernatant discarded, and the crude pellets were rinsed by partial resuspension in ice-cold 750 µL ChIP Lysis Buffer, and spun again for 15 min. For sonication, pellets were thoroughly resuspended with gentle probe sonication in fresh ChIP Lysis Buffer at 0°C, and chromatin was sheared by 60 cycles of a 30 sec burst followed by a 60 sec rest in a Bioruptor XL (Diagenode) at high power with circulating chilled water at 2°C. Debris was pelleted by centrifugation for 5 min at 23 K rpm at 0°C, and the soluble chromatin supernatant was aliquoted on ice, flash frozen in liquid nitrogen, and stored at −80°C. Chromatin shearing adequate for fine resolution analysis was confirmed by DNA gel electrophoresis of de-crosslinked and purified samples, and showed that the average fragment lengths were approximately 200 bp.

ChIP was carried out using 1 µg affinity purified anti-histone H3 C-terminus or anti-Myc (Mouse MAb 4A6, Upstate Biotechnology) or 5 µL anti–Spt16 serum rabbit polyclonal antibodies (Tim Formosa, University of Utah) pre-bound to approximately 3×10^7^ anti-Rabbit IgG-coated magnetic beads (Invitrogen) per reaction. Immunoprecipitations contained chromatin with 500 µg protein content measured by BCA assay, beads, 1.5 mg/mL BSA, all in ChIP Lysis buffer as above, except with 0.01% SDS and 0.5 mM MgCl_2_ instead. Beads were washed for 5 min at 4°C twice with ChIP Lysis buffer as above, except with no SDS, twice with ChIP Lysis buffer as above, except with 500 mM NaCl and no SDS, and twice with ChIP Wash Buffer [48]. ChIP reactions were eluted from beads and de-crosslinked using Chelex-100 according to [49]. Aliquots of each input total chromatin corresponding to 25 µg protein content were de-crosslinked in parallel. Occupancy was measured by qPCR using primers for the indicated target and reference locations. SYBR-Green containing qPCR reactions with 1/4^th^ to 1/20^th^ dilution of the ChIP reaction and 1/200^th^ to 1/1000^th^ dilution of the input total chromatin were carried out in a Lightcycler LC480 (Roche), and were then quantified by also preparing a dilution set of the input total chromatin reactions analyzed in parallel to generate a standard curve, and calculating each target's relative concentration from the reaction's Cq using the manufacturer software. Levels of occupancy were compared between samples by first calculating a ChIP signal by dividing the relative concentration of a target in the ChIP reaction by that of the input chromatin to obtain a fraction or % IP value, then normalizing each ChIP signal to that for the IGR-I gene-free reference region on chromosome I [50]. Error bars for each point represent the propagated error from two to three PCR reactions each for the target sequence in the ChIP reaction and input, as well as for the reference sequence in the ChIP reaction and input, according to [51], [52]. Thus, a value of 1.0 on the y-axis in the figures corresponds to Histone H3 association, and thus inferred nucleosome occupancy equal to that of the non-expressed reference region, except where normalized to untreated samples.

RNA was prepared from corresponding 15 mL culture aliquots by adding Ethanol to 70%, then flash freezing the cell suspension in liquid nitrogen, and storing at −80°C for processing. RNA was isolated from frozen cell pellets by extraction with hot acid phenol, followed by precipitation through 5.7 M CsCl modified from [53]. Gene expression was analyzed by RT-qPCR, using randomly primed MMLV-RT (Promega) cDNA synthesis reactions containing 1 µg RNA. SYBR-Green containing qPCR reactions with 1/200^th^ dilution of the RT reaction and using gene-specific primers (see Table S2) in a Lightcycler LC480 (Roche) were then quantified using pooled cDNA dilutions to generate standard curves as for the ChIP analysis [54]. Levels of *PDR* gene expression were compared between samples by normalizing each signal to the *RPR1* internal reference transcript, or in certain indicated experiments to the *RDN25* or the *SCR1* internal reference.

## Results

### The Pdr1 transcription factor interacts with Gal11 and FACT

In order to identify proteins interacting with the Pdr1 transcriptional activator, we used the Pdr1 activation domain immobilized as a GST fusion protein for affinity chromatography, potentially bypassing any xenobiotic binding requirement on the activation domain. Specifically bound proteins eluting from the matrix were identified by mass spectrometry, and included both the Spt16 and Pob3 subunits of FACT, as well as the Gal11 subunit of Mediator (Fig. S1). We have previously shown that the KIX domain of Gal11 interacts with Pdr1, and that *gal11* mutations diminish the PDR response [22]. Notably, mutations in some other Mediator components did not have this same effect [23]. Thus, identification in parallel of a known Pdr1-interacting factor necessary for PDR response serves as a positive control for the FACT-Pdr1 association, and suggests that recovery of FACT is physiologically significant.

### FACT mutations affect antifungal sensitivity, and are additive with Mediator mutations

**Figure 1:**
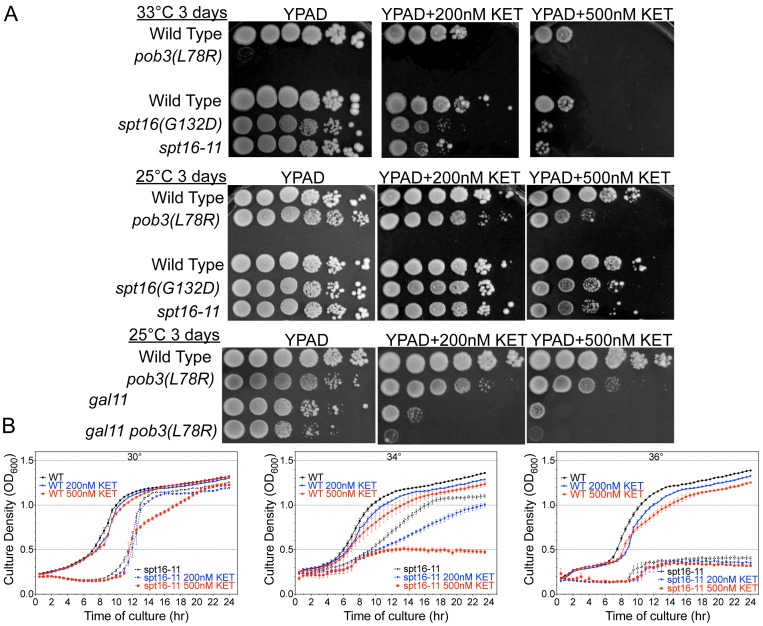
Mutations in FACT sensitize cells to the antifungal ketoconazole. A) Serial dilutions of DY150 (WT), DY5628 (*gal11*), DY6189 (*spt16(G132D*)), DY7230 (*spt16-11*), DY7379 (*pob3(L78R*)), or DY14202 (*gal11 pob3(L78R*)) were identically spotted onto YPAD agar plates lacking or containing 200 or 500 nM ketoconazole, grown under the indicated conditions, and then photographed. B) Dilute cultures of DY150 (WT) and DY8107 (*spt16-11*) were incubated in quadruplicate at the indicated temperatures in the absence or presence of 200 nM or 500 nM ketoconazole while OD600 was measured at 30 min. intervals and plotted as a function of incubation time. Error bars indicate the SEM of the four duplicate culture readings at each time point.

FACT has been shown to play a role in transcriptional activation at promoters [37], [46], [47] and to interact directly with the transcription factor SBF [55]. The Pdr1 – FACT interaction might also be direct, or it could involve intermediary bridging factors. Consistent with the latter possibility, we were unable to effectively co-immunoprecipitate Pdr1 with FACT. Previous work has shown that *gal11* mutations confer sensitivity to xenobiotic drugs [22], so to test the relevance of FACT in *PDR* gene activation we asked whether FACT mutations have a similar effect by plating serial dilutions onto plates containing ketoconazole, an antifungal drug and inducer of *PDR* gene expression reviewed in [2], [56]. The FACT mutants show some growth defect in the presence of ketoconazole, and the FACT *gal11* double mutants show a significant additive growth defect (Fig. 1). This suggests that Gal11 and FACT act in parallel on the same process in PDR.

### PDR expression and Mediator recruitment respond rapidly to antifungal exposure

**Figure 2:**
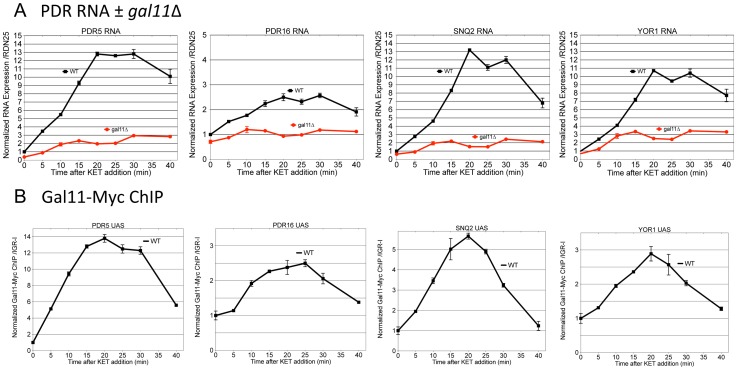
PDR genes are rapidly induced by drug treatment, in a Gal11 dependent process. A) RT-qPCR analysis of several PDR gene RNAs from ketoconazole treated cultures of *gal11* and FACT mutant strains: DY6129 (WT, black lines) vs. DY4257 (*gal11*, red lines) were grown to mid-log density at 25°C, temperature shifted to 37°C for 1 hr, 40 µM ketoconazole was added, and then samples were taken at the indicated times. RNA was isolated and RT-qPCR performed using primers targeting the transcribed regions of *PDR5*, *PDR16*, *SNQ2*, and *YOR1*, with results plotted as fold relative to the *RDN25* reference transcript. B) ChIP with anti-Myc antibodies directed against C-terminally Myc tagged Gal11, from the same DY 6129 (WT) cultures as in A, using primers targeting the full UAS regions of several PDR genes encompassing the PDRE sequences for *PDR5*, *PDR16*, *SNQ2*, and *YOR1*. ChIP signals are expressed as fold enrichment relative to the intergenic IGR-I reference target, and are normalized to non drug-treated samples. Error Bars represent the SD of three replicate qPCR reactions. Data presented are representative of two to three independently replicated experiments. See Table S2 for primer sequences.

**Figure 3:**
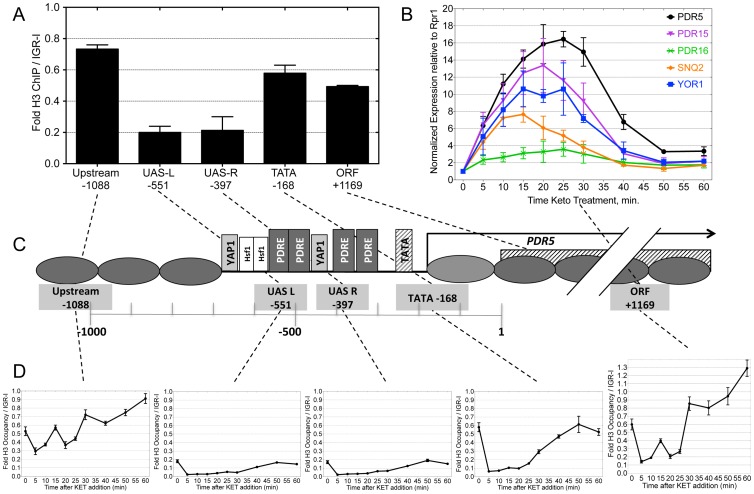
Nucleosome depletion and re-deposition across *PDR5* during induction by exposure to ketoconazole. A) Histone H3 ChIP was used to measure nucleosome occupancy at the indicated regions relative to the gene-free IGR-I region across the *PDR5* promoter and ORF in a DY150 (WT) strain grown @ 30°, using these primers: *PDR5* Upstream (F2710- F2711), *PDR5* UAS-L (primers F2460- F2712), *PDR5* UAS-R (primers F3229- F3230), *PDR5* TATA (primers F2488-F2489), and *PDR5* ORF (primers F3198-F3199). Error Bars represent the SD for three biological replicate cultures. Results show the relatively depleted region encompassing the Pdr1 and Yap1 binding sites in the promoter as shown in C, with higher occupancy elsewhere. B) RT-qPCR analysis of several PDR gene RNAs (using *PDR5*, *PDR15*, *PDR16*, *SNQ2*, and *YOR1* ORF primer sets) from DY150 (WT) cells treated with a 60 min. timecourse of ketoconazole, plotted as fold relative to the RPR1 reference transcript. Gene expression was normalized to the basal levels prior to treatment. Error Bars represent the data range of duplicate RT and qPCR reactions. C) Diagram of the *PDR5* promoter and ORF, showing relative positions of nucleosomes (ovals) based on data from [57]–[59], PDRE (potential Pdr1 binding sites) and Yap1 sites, the TATA, TSS, and ATG, and PCR target amplicons. D) Histone H3 ChIP was used to measure nucleosome loss and re-deposition at the indicated regions relative to IGR-I across *PDR5* just before and at intervals for 1 hour after addition of ketoconazole to DY150 (WT) cultures. Values are calculated as in A. Error Bars represent the SD of three replicate qPCR reactions. Data presented in 3B and 3C are typical of experiments independently repeated at least six times. See Table S2 for primer sequences.

*PDR* genes are expressed at modest levels in the absence of exogenous xenobiotic agents, but are rapidly induced to high levels following drug exposure (Fig. 2A and Fig. 3B) [17], [22]. In order to more precisely characterize the role of Gal11 in *PDR* gene activation, we treated logarithmically growing cells, wild type and *gal11* mutants, with high dose (40 µM) ketoconazole, and harvested samples periodically over 40 min for RNA analysis and for Gal11 ChIP to measure Mediator recruitment. We observed a marked defect in the normally rapid *PDR* gene induction in *gal11* mutant strains (Fig. 2A) and a similar defect in multiple FACT mutants with different growth conditions (Fig. S2 and Fig. S3). ChIP experiments show Gal11 is rapidly recruited to the *PDR* promoters following ketoconazole exposure (Fig. 2B), with the kinetics of Gal11 binding roughly correlating with gene activation. These experiments support the idea that both Gal11 and FACT are required for *PDR* gene activation, with the kinetics of Mediator recruitment suggesting a direct role in activation.

### Transcriptional response and histone occupancy of *PDR* genes is rapid and dynamic

In order to understand the role of FACT and chromatin in the PDR response, we first analyzed nucleosome occupancy by using Histone H3 ChIP assays in the promoter and coding regions in the absence of toxin treatment. For our chromatin studies we focused on the canonical Pdr1 target *PDR5*, encoding a prominent drug efflux pump, and determined H3 binding in the uninduced state. We used five sets of PCR primers, for the far upstream region, two parts of the Upstream Activating Region (UAS-L and UAS-R), the TATA region, and within the transcribed ORF (see diagram in Fig. 3C). The ChIP experiments in Fig. 3A clearly show that the *PDR5* UAS is within a Nucleosome Depleted Region (NDR), consistent with whole-genome analyses of histone occupancy [57]–[59]. (As described in Methods, for all histone H3 ChIP assays a value of 1 on the y-axis corresponds to the inferred nucleosome occupancy seen at a gene-free reference region on chromosome I.).

We first analyzed the kinetics of the transcriptional response and changes in chromatin structure in wild type cells grown at the standard temperature of 30°C. Cells were treated with ketoconazole and samples were taken periodically over 60 minutes for both RNA analysis and for histone H3 ChIP to measure nucleosome occupancy. RT-qPCR was used to measure mRNA levels for five genes of the PDR regulon, including *PDR5*, and the results show rapid induction, visible at the first time point (5 min), a peak at 15 to 25 min, and a return to baseline at 40 to 50 min (Fig. 3B). Normalizing the data for each gene to transcript levels in untreated cells, induction levels of 4 to 16 fold are seen.

The time course ChIP experiments (Fig. 3D) show an immediate depletion of histones at 5 min following drug addition in the TATA and ORF regions, and even within the UAS which contains sufficiently low histone occupancy to be described as an NDR. Surprisingly, this histone loss is also seen upstream of the UAS, suggesting that effects on chromatin from drug binding by the Pdr1 factor can be propagated bi-directionally. After the initial rapid loss, histone H3 signal begins to return to the upstream regions first, with detectable repopulation by 10 minutes, while the signal remains transiently depleted across the entire promoter and transcription unit. Nucleosome loss across the promoter and coding region persists until about 25 minutes, and returns to the full basal occupancy conditions at 40 to 50 minutes, even though drug is still present. Note that nucleosome binding is inversely correlated with *PDR5* transcription.

### Altered chromatin dynamics at *PDR5* in FACT mutant strains

We next asked how FACT defects affect the dynamics of chromatin alterations at *PDR* genes during activation and repression. Most FACT mutations are temperature sensitive for growth [31], and many studies, including those in Fig. S2, utilize a temperature shift protocol in order to reveal FACT defects. However, such procedures also induce a heat shock response, and this will lead to activation of many *PDR* genes because most of these genes, including *PDR1* and *PDR3*, also contain binding sites for the heat shock-induced Hsf1 and/or Yap1 factors adjacent to the Pdr1 binding sites [60], [61] (also see Fig. 3C). For this reason we wanted to avoid a sudden change in temperature in our studies. To this end, growth curves were generated for wild type and *spt16-11* (aa T828I, P859S) mutant strains grown continuously in liquid culture at various temperatures in the presence and absence of ketoconazole (Fig. 1B). At the semi-permissive temperature of 36°C the *spt16-11* mutant grows very poorly, while at 34°C the *spt16-11* strain displays relatively normal growth characteristics in the absence of added drug. We decided to use the *spt16-11* mutant grown at 34°C in subsequent experiments; importantly, under these conditions ketoconazole inhibits growth in a dose-dependent manner (Fig. 1B, middle panel). In our experiments, the wild type and *spt16-11* strains were cultivated at 34°C continuously for at least 12 hours to attain mid-log density before addition of drug, allowing temperature adaptation and thus avoiding any heat shock effects.

**Figure 4:**
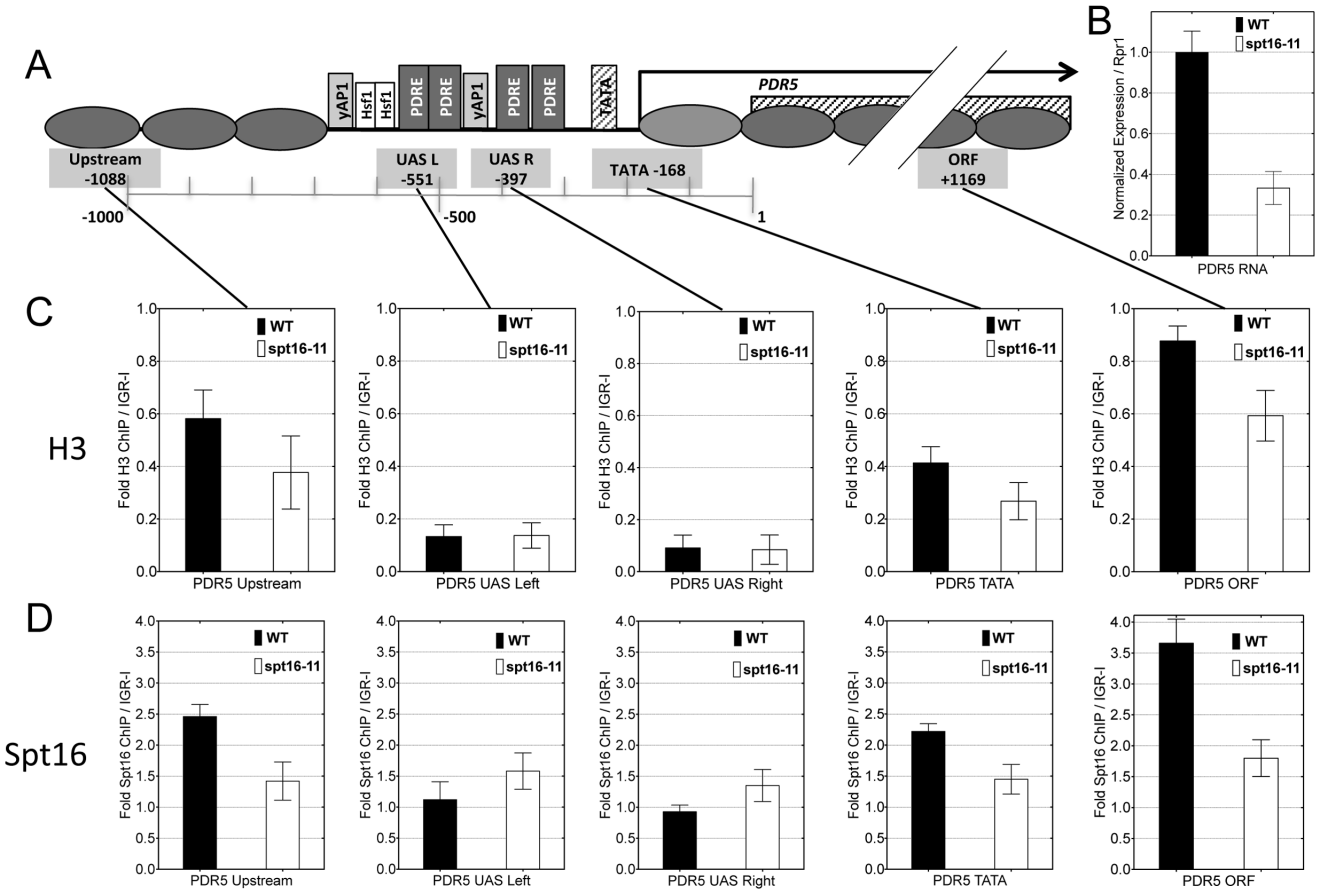
*spt16-11* alters histone binding across *PDR5*. A) Diagram of *PDR5* as in Fig. 3. B) RT-qPCR analysis of *PDR5* RNA from non-drug treated DY150 (WT, filled bars) or DY8107 (*spt16-11*, open bars) cells, both grown at 34° and normalized to *RPR1* reference transcripts, and plotted relative to WT levels. C) ChIP analysis using antibodies against histone H3 at the indicated locations in *PDR5* from the same non-drug treated WT or *spt16-11* strains as above, normalized to an intergenic region on chromosome I (IGR-I) as a reference control. D) ChIP analysis using antibodies against Spt16 similarly to C. For all graphs, error bars represent the SD for three biological replicate cultures.

We first studied wild type and *spt16-11* mutant cells grown at 34°C in the absence of drug, measuring *PDR5* mRNA levels by RT-qPCR and examining *PDR5* chromatin architecture by histone H3 ChIP. Basal expression of *PDR5* is reduced about 3-fold in the *spt16-11* mutant (Fig. 4B). Similar mild reductions are seen at several other *PDR* genes in *spt16-11* (e.g., *PDR16* and *SNQ2*) and with several other mutant FACT alleles (Fig. S2 and Fig. S3), indicating this effect is not FACT allele-specific, nor due to the normalization methods. Chromatin structure at *PDR* genes in wild type cells at 34°C is similar (Fig. 4C) to that seen at 30°C (Fig. 3A), as evidenced by histone H3 ChIP signals. In *spt16-11* cells at 34°C, the NDR is still present at the *PDR5* promoter, but the histone H3 ChIP signals in more highly occupied locations are somewhat reduced (Fig. 4C), suggesting FACT-dependent alterations to chromatin architecture in the absence of toxin. To determine whether the nucleosome occupancy levels at *PDR5* are directly due to FACT, Spt16 ChIPs were performed in uninduced wild type or *spt16-11* cells (Fig. 4D). FACT occupancy mirrors that of nucleosomes at this gene, with Spt16 ChIP signals relatively increased at those locations with higher histone H3 ChIP signals, but with signals at or below background levels in the NDR region of the *PDR5* UAS. To address the specificity of the Spt16 ChIP signals, the *spt16-11* negative control ChIP (Fig. 4D, open bars) was performed because the specific enrichment of wild type FACT at any particular gene relative to control is usually mild, probably due to FACT binding activity throughout the genome. Inactive mutant FACT protein levels are probably quite reduced at this semi-permissive temperature in mutant cells, based on the reduced levels seen in Western blots at the non-permissive temperature of 37°C [62], and loss of FACT function under these conditions has been observed in other assays [63]. The low enrichment values for FACT binding across *PDR5* in the *spt16-11* strain grown at 34°C thus define ChIP background signals. However, the small reductions from loss of FACT binding and function in both expression (∼3-fold) and histone occupancy in non-drug treated cells are unlikely to account for the observed defects in the PDR phenotype.

**Figure 5:**
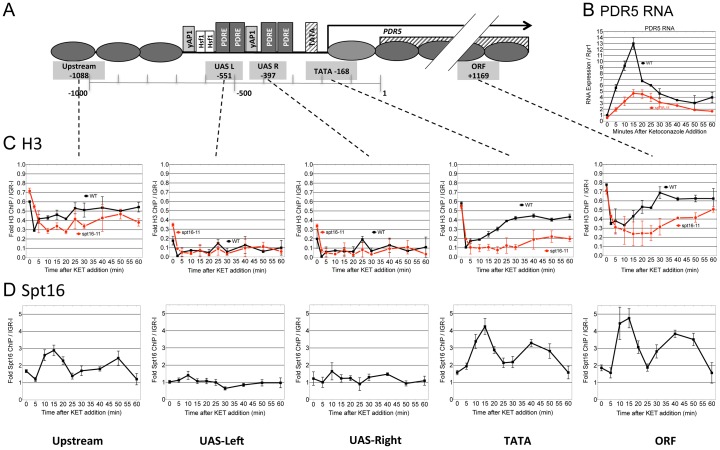
Kinetics of induction and nucleosome re-deposition at *PDR5* correspond to FACT occupancy, and are defective in a FACT mutant. DY5699 (WT, black lines) and DY8107 (*spt16-11*, red lines) cultures were grown at 34°C and treated with ketoconazole, with samples collected every 2.5, 5, or 10 min. for ChIP and RNA during 60 minutes of exposure. RNA (5B) and ChIP (5C and 5D) analyses were as in Fig. 3 and Fig. 4, at locations in *PDR*5 shown in 5A. C) H3 ChIP across *PDR5* during drug induction, showing rapid nucleosome loss and differences in repopulation. D) Chromatin samples from the same collection as those in Fig. 5C were analyzed by ChIP using antibodies against Spt16 for FACT recruitment to *PDR5*. Data are reported as Fold ChIP signal relative to the gene-free IGR-I region. Error bars represent the data range for two biological replicate cultures.

To address the hypothesis that the effects of FACT loss are more important to dynamic changes in PDR induction upon drug exposure rather than basal expression, we next added 40 µM ketoconazole to wild type and *spt16-11* cells grown continuously at 34°C. *PDR5* mRNA was measured by RT-qPCR (Fig. 5B) and histone H3 occupancy across *PDR5* was assayed by ChIP (Fig. 5C), over a longer period and with more frequent sampling for finer resolution. *PDR5* induction occurs somewhat faster in wild type cells at this temperature. Importantly, the amplitude of *PDR5* RNA accumulation and the rate of increase were both lowered in the *spt16-11* strain (Fig. 5B), and similar reductions and delays were also seen at other *PDR* genes (Fig. S3, which also includes the same data from Fig. 5B for comparison), with the time of peak mRNA abundance delayed by at least 10 min relative to WT. The transcription of *PMA1*, encoding the membrane H+ ATPase required for PDR, but not regulated by Pdr1, [64], [65] did not show a similar profile of FACT-dependent induction, indicating that this burst of transcription is not a general response (Fig. S3). Time course gene expression experiments with the other mutant FACT alleles also show defects in *PDR* gene induction in response to xenobiotic drugs (Fig. S2). In contrast, a *gal11* mutation affecting Mediator function causes a much more severe defect in *PDR* gene induction (Fig. 2A) [22].

Histone H3 chromatin binding measurements upon ketoconazole induction throughout the *PDR5* promoter and ORF were then undertaken. These time course experiments showed a rapid loss of histone H3 ChIP signal upon ketoconazole addition in the upstream, TATA and ORF regions, with minimum occupancy at 2.5 to 5 min after drug treatment in wild type cells (Fig. 5C). Importantly, this rapid loss was observed in both wild type and *spt16-11* cells, notwithstanding the transcriptional induction defects observed in *spt16-11* at the same time, although there may be a slightly delayed eviction in the absence of FACT. This analysis concluded that nucleosome occupancy was rapidly restored to pre-induction levels in wild type cells. However, although FACT mutants exhibited nucleosome loss with kinetics similar to wild type cells, the *spt16-11* mutant exhibited a marked defect in the ability to repopulate chromatin to the pre-induction level of histone occupancy compared to wild type. Histone H3 association was still deficient 60 min following drug addition in the FACT mutant, while in wild type, nucleosomes were fully repopulated by 30 min. This same overall pattern of nucleosome binding dynamics is reproducibly seen for several genes in the *PDR* regulon (Fig. S4).

ChIPs were performed with anti-Spt16 antibody to measure FACT recruitment to *PDR5* during the time course described for Fig. 5B and 5C. As shown in Fig. 5D, there is a low amount of FACT binding to the ORF before induction, but FACT occupancy increases during the initial chromatin disassembly phase that accompanies gene induction (10 min). Substantially more FACT binding is seen after gene expression has ended, coinciding with the time of nucleosome repopulation (30 min). Relatively little FACT is recruited to the NDR region at the UAS.

## Discussion

Transcriptional activation of the *PDR* genes is unusual because the Pdr1 transcription factor is always promoter-bound, even in the absence of inducers. Upon binding to xenobiotic agents the large carboxyl-terminal domain harboring the activation domain of Pdr1 changes in unknown ways to facilitate gene activation. This transcriptional response requires the Gal11 subunit of Mediator, and here we show that there is rapid recruitment of Gal11 to *PDR* gene promoters coincident with the sudden burst of gene activation.

Mass spectroscopy of Pdr1 interacting proteins detected the Spt16 and Pob3 subunits of the FACT chromatin reorganizing complex, and we find that FACT affects *PDR* gene expression in unexpected ways. FACT mutants show an increased sensitivity to xenobiotic drugs and a decrease in both basal and induced *PDR* gene expression. At some promoters, sequence-specific DNA-binding factors recruit FACT, which can then facilitate nucleosome eviction at promoters as a prelude to gene activation [46], [47], [66]. We expected a similar scenario at the *PDR* genes. However, the promoters of these genes have regions spanning the PDREs that are already largely nucleosome free without stimulation, in addition to constitutive occupancy by Pdr1, and thus the promoters do not require nucleosome eviction before their rapid transcriptional response. We could not detect additional FACT recruited to *PDR* gene control elements during induction, possibly due to the reduced presence of its nucleosomal substrate there, but only to transcribed regions, consistent with FACT's proposed roles in elongation. Further work is needed to determine if the FACT binding signals observed in the proximal promoter TATA region of *PDR5* could be distinct from that in the elongation region, or is a limitation of the resolution of the ChIP assay. It is notable that FACT is recruited to the ORF in two phases: The first coincides with nucleosome loss as transcription is induced, and the second phase occurs concurrent with nucleosome reassembly following conclusion of transcription. This biphasic profile of recruitment is similar to that seen at *CLN* genes during cell-cycle induction in G1 [55]. The major defect observed in FACT mutants at *PDR* genes was a marked delay in repopulation of nucleosomes after transcription ceases in this second phase.

This defect in nucleosome repopulation is consistent with FACT's proposed roles in both nucleosome disassembly and reassembly [29]. FACT mutant phenotypes of cryptic promoter activation [40], [43], failure to rebuild properly repressive chromatin [67], and enhanced rates of histone replacement [68] support models in which FACT is required for nucleosome reassembly. Experiments presented in this work were carried out such that the rapid kinetics of the PDR gene response were able to be measured, and moreover the effects of FACT on the dynamic processes of nucleosome disassembly and reassembly during a natural cycle of induction and resolution could be directly observed in chromatin composed of WT histones at native levels of abundance, in contrast to previous approaches. Most surprisingly, FACT mutations reduce both basal expression of *PDR* genes as well as the rate of gene induction, even though chromatin looks normal in the gene promoter. Why does a failure to reassemble nucleosomes over *PDR* genes lead to reduced gene expression and a drug resistance defect, rather than to a derepressed transcriptional status? While there is a somewhat delayed return to uninduced levels at some *PDR* genes in FACT mutant cells, it seems likely that the observed transcriptional defects in FACT mutants are probably due to aberrant reassembly of chromatin following DNA replication in drug-free cells, as the *PDR* genes have not been induced for many generations in our experimental setup. Further analysis of the role of FACT in determining the nature of *PDR* gene chromatin will be required.

PDR in fungi, and MDR more generally, occur in pathogenic conditions and must be managed for successful clinical outcomes. Understanding the roles of coactivators and modifiers of chromatin at ABC transporter genes define prominent molecular targets, which may allow the design of adjunct drug therapies targeting these proteins that would inhibit resistance to antifungals or chemotherapeutics. Moreover, drugs which interfere with factors that modify chromatin, with FACT among them, have been explored as chemotherapeutic agents [69], [70], however these may have unintended consequences for altering ABC gene expression, both by activating MDR, as well as interfering with chromatin regulators at those genes, and therefore enhancing resistance in unexpected ways. A thorough understanding of all factors involved in MDR gene regulation will allow rational intervention design to clinically manage this phenomenon.

## Supporting Information

Figure S1 **Yeast FACT and Gal11 bind to the Pdr1 activation domain.**
(PDF)

Figure S2 **Altered** ***PDR5*** **expression kinetics observed in other FACT mutants.**
(PDF)

Figure S3 ***SPT16*** **mutation reduces and/or delays the kinetics of antifungal induced expression of multiple** ***PDR*** **genes.**
(PDF)

Figure S4 **Many genes in the PDR regulon show histone binding alterations during ketoconazole induction.**
(PDF)

Table S1 Yeast Strains and Plasmids.
(PDF)

Table S2 Oligonucleotide List.
(PDF)
