# Supplementary material for: A Role for FACT in Repopulation of Nucleosomes at Inducible Genes
Source: PLoS One. 2014 Jan 2;9(1):e84092. doi: 10.1371/journal.pone.0084092 (PMC3879260; doi:10.1371/journal.pone.0084092)
Supplement: Figure S1 — Yeast FACT and Gal11 bind to the Pdr1 activation domain. (PDF) [file pone.0084092.s001.pdf]

Unique	Total	AVG XCorr	Reference	Gene Symbol
42	65	3.488598479	YGL207W	*SPT16*
19	52	3.529628850	YOL051W	*GAL11*
11	15	3.100746679	YML069W	*POB3*

Supplemental Figure S1. Yeast FACT and Gal11 bind to the Pdr1 activation domain. Tabular representation of selected top Pdr1 AD interacting proteins identified by tandem mass spectrometry. FACT subunits Spt16 and Pob3 were among the most abundantly Pdr1 AD associated proteins as assessed by number of peptides and percent sequence coverage, along with the previously identified Mediator subunit Gal11, which served as a positive control in the experiment.
