# Supplementary material for: A Role for FACT in Repopulation of Nucleosomes at Inducible Genes
Source: PLoS One. 2014 Jan 2;9(1):e84092. doi: 10.1371/journal.pone.0084092 (PMC3879260; doi:10.1371/journal.pone.0084092)
Supplement: Figure S2 — Altered PDR5 expression kinetics observed in other FACT mutants. (PDF) [file pone.0084092.s002.pdf]

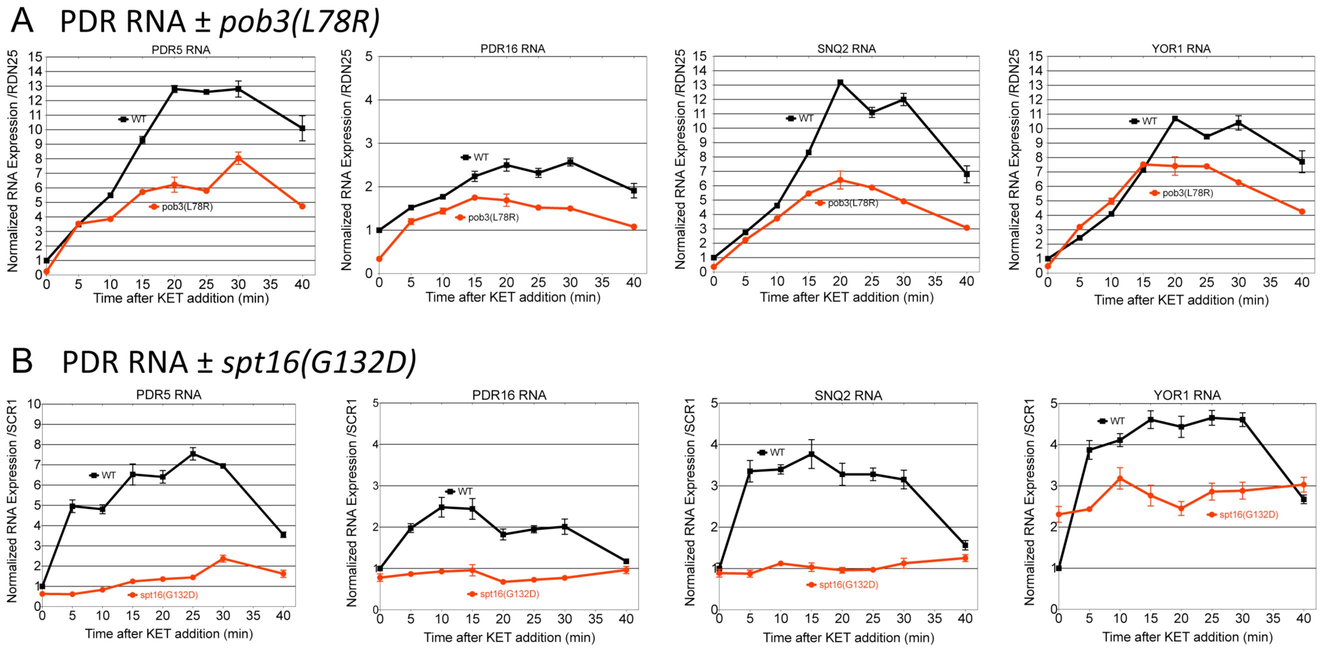

Supplemental Figure S2. Altered *PDR5* expression kinetics observed in other FACT mutants.

RT-qPCR analysis of several PDR gene RNAs from ketoconazole treated cultures: In A) DY150 (WT, black lines) vs. DY7379 (*pob3(L78R)*, red lines) were analyzed as in Fig 2. RT-qPCR results are plotted as fold relative to *RDN25* reference transcripts. B) The same analysis as in A), but using DY150 (WT, black lines) vs. DY6189 (*spt16(G132D)*, red lines) prepared and analyzed as in Figure 2, except that cells were grown at 30°C, then shifted to 37°C for 1 hr prior to drug treatment, and with gene expression levels measured relative to *SCR1* reference transcripts. Gene expression was normalized to basal levels prior to treatment, and expressed as fold relative to untreated WT samples. Error bars represent the SD of three replicate qPCR reactions.
