# Supplementary material for: A Role for FACT in Repopulation of Nucleosomes at Inducible Genes
Source: PLoS One. 2014 Jan 2;9(1):e84092. doi: 10.1371/journal.pone.0084092 (PMC3879260; doi:10.1371/journal.pone.0084092)
Supplement: Figure S3 — SPT16 mutation reduces and/or delays the kinetics of antifungal induced expression of multiple PDR genes. (PDF) [file pone.0084092.s003.pdf]

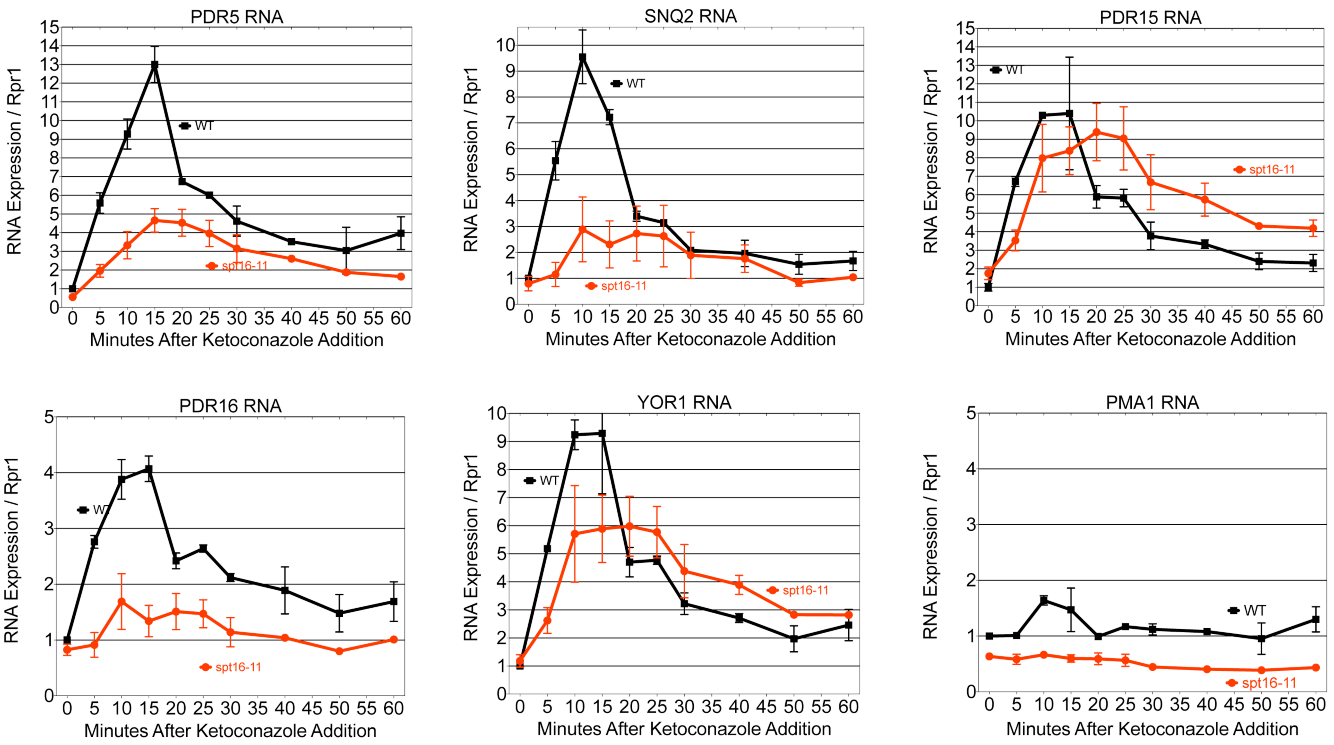

Supplemental Figure S3. ***SPT16*** **mutation reduces and/or delays the kinetics of antifungal induced expression of multiple** ***PDR*** **genes.**

RT-qPCR analysis of samples collected as shown in Fig 5 from DY5699 (WT, black lines), or DY8107 (*spt16-11*, red lines) was performed on multiple genes of the PDR regulon, using *PDR5*, *PDR15*, *PDR16*, *SNQ2*, and *YOR1* ORF primer sets, with *PDR5* data from Figure 6 included for comparison. Expression of *PMA1*, is included as a control, showing no drug-dependent induction kinetics. Error bars represent the data range from the calculated SD for two replicate RT reactions analyzed by two replicate PCR reactions. Data presented are typical of experiments independently repeated at least six times.
