# Supplementary material for: A Role for FACT in Repopulation of Nucleosomes at Inducible Genes
Source: PLoS One. 2014 Jan 2;9(1):e84092. doi: 10.1371/journal.pone.0084092 (PMC3879260; doi:10.1371/journal.pone.0084092)
Supplement: Figure S4 — Many genes in the PDR regulon show histone binding alterations during ketoconazole induction. (PDF) [file pone.0084092.s004.pdf]

Supplemental Figure S4

Repopulation of nucleosomes by FACT

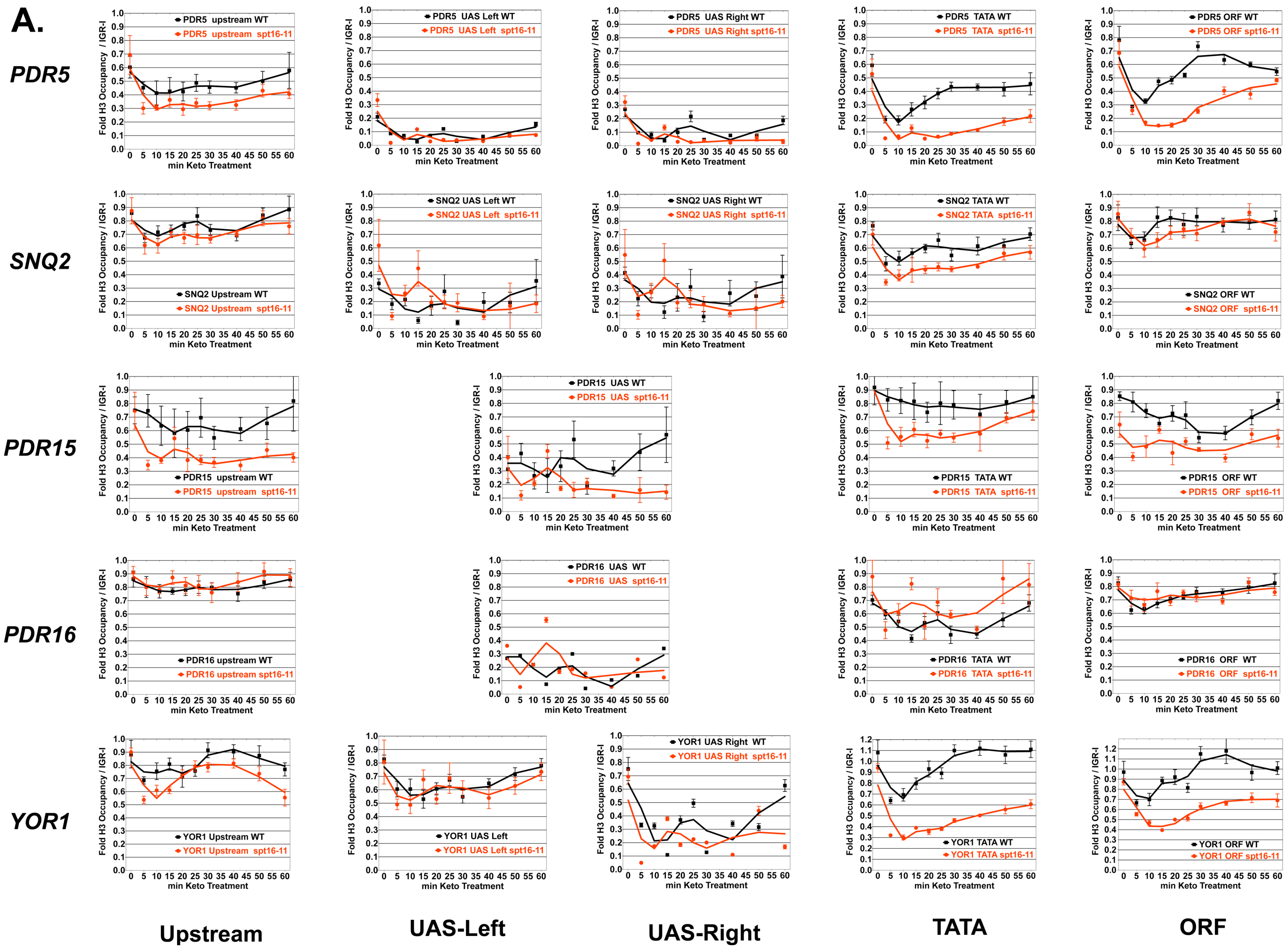

Supplemental Figure S4

Repopulation of nucleosomes by FACT

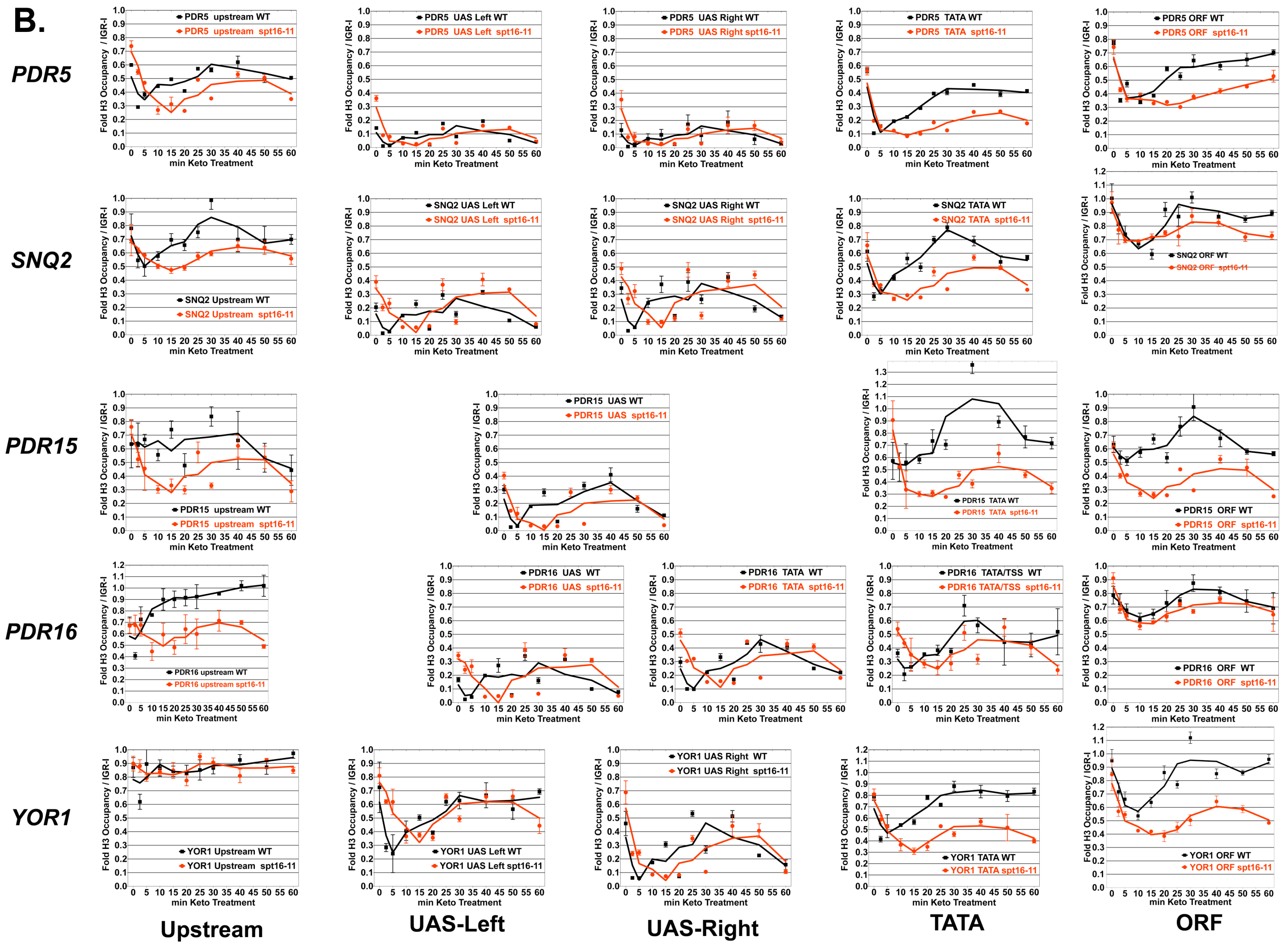

Supplemental Figure S4. Many genes in the PDR regulon show histone binding alterations during ketoconazole induction. DY5699 (WT), and DY8107 (*spt16-11*) cultures were grown and prepared as in Fig 5. ChIP analysis was as in Fig 3 and Fig 4, using primers for subdivided regions of each PDR gene's promoter and coding region (See Table 2). Lines (black for WT, red for *spt16-11*) are 2nd order smoothing of 2 nearest neighbor data points. Error bars for each point represent the cumulative error from two PCR reactions each for the target sequence in the ChIP reaction and input, as well as for the reference sequence in the ChIP reaction and input. Two fully independent experiments (A and B) showing the same effects of *spt16-11* on nucleosome occupancy are shown.
