# Supplementary material for: A Role for FACT in Repopulation of Nucleosomes at Inducible Genes
Source: PLoS One. 2014 Jan 2;9(1):e84092. doi: 10.1371/journal.pone.0084092 (PMC3879260; doi:10.1371/journal.pone.0084092)
Supplement: Table S1 — Yeast Strains and Plasmids. (PDF) [file pone.0084092.s005.pdf]

Supplemental Table S1. Yeast Strains and Plasmids.

Strains

DY150	MAT**a** *ade2 can1 his3 leu2 trp1 ura3*
DY4257	MAT**a** *gal11::LEU2 ade2 can1 leu2 trp1 ura3*
DY5628	MAT**a** *gal11::LEU2 ade2 can1 his3 leu2 lys2 trp1 ura3*
DY5699	MAT**a** *ade2 can1 his3 leu2 trp1 lys2 met15 ura3*
DY6189	MAT**a** *spt16(G132D) ade2 can1 his3 leu2 trp1 ura3*
DY7230	MAT**a** *spt16-11(T828I, P859S) ade2 can1 his3 leu2 lys2 met15 ura3*
DY7379	MAT**a** *pob3(L78R) ade2 can1 his3 leu2 lys2 met15 trp1 ura3*
DY8107	MAT**a** *spt16-11 ade2 can1 his3 leu2 lys2 met15 trp1 ura3*
DY12746	MAT**a** *Gal11-Myc::HIS3MX pob3(L78R) ade2 can1 his3 leu2 lys2 trp1 ura3*

GST Plasmids

The GST-PDR1-AD, GST-Gal11, and GST-Gal11(E53A) plasmids have been previously described [1].

References

1. Thakur JK, Arthanari H, Yang F, Pan S-J, Fan X, et al. (2008) A nuclear receptor-like pathway regulating multidrug resistance in fungi. Nature 452: 604-609.
