# Supplementary material for: A Role for FACT in Repopulation of Nucleosomes at Inducible Genes
Source: PLoS One. 2014 Jan 2;9(1):e84092. doi: 10.1371/journal.pone.0084092 (PMC3879260; doi:10.1371/journal.pone.0084092)
Supplement: Table S2 — Oligonucleotide List. (PDF) [file pone.0084092.s006.pdf]

Supplemental Table S2. Oligonucleotide List.

ChIP-qPCR oligos

F1399	*IGR-V*	GGCTGTCAGAATATGGGGCCGTAGTA
F1400	*IGR-V*	CACCCCGAAGCTGCTTTCACAATAC
F1416	*IGR-I*	GTTTATAGCGGGCATTATGCGTAGATCAG
F1417	*IGR-I*	GTTCCTCTAGAATTTTTCCACTCGCACATTC
F2460	*PDR5* UAS	CAACGATTATCACGACACAAC
F2461	*PDR5* UAS	GCACATTACCATAAGTAGGAA
F2462	*PDR5* ORF	TACCGCTTTGGAATTTATTCG
F2463	*PDR5* ORF	GCATATCTTTGTTCAGGGTTC
F2464	*PDR16* UAS	GAAATAGCAGTAACCCAACTC
F2465	*PDR16* UAS	CAAATAGAGTCGGGTGGAATG
F2466	*PDR16* ORF	GTCGAGAACGAATCAGGTAAG
F2467	*PDR16* ORF	GGTACTTTAGGAACATCAGGG
F2470	*SNQ2* ORF	GTATCTGGTGGTGAACGTAAG
F2471	*SNQ2* ORF	CTTGGAGGACACAAATAACCC
F2472	*YOR1* UAS	GGTTCTCTACTAAATGCTTGC
F2473	*YOR1* UAS	GGTGTTAAATCATGTCCGTC
F2474	*YOR1* ORF	TTCCTAACGGTAAAGTGACTTC
F2475	*YOR1* ORF	GATGTTCGCAGCAATTCTAAA
F2482	*SNQ2* UAS	CTTCACGCCAGACTATGTATG
F2483	*SNQ2* UAS	GTTGATGTTGCGTAGTCACTA
F2488	*PDR5* TATA	AACGTAATCTGAGCAATACAAAC
F2489	*PDR5* TATA	CGAAAGTTCCTAGTTGCCAAT
F2710	*PDR5* Upstream	CTGAAATGTGACACAGTAAAGTCCGTC
F2711	*PDR5* Upstream	CCAGAGAACGCTTCCTTTGTATGA
F2712	*PDR5* UAS Left Lower	GCAGTCTTAAGTTTCAGTCCACT

F2713	*PDR5* UAS Right Upper	ACAACATTTTCAGATTTACTAAGA
F2714	*PDR15* UAS Upstream	AAACGAGAGATAGACAATAAGCGAGAG
F2715	*PDR15* UAS Upstream	TTATCTGGGTATTTGCGTGTGG
F2716	*PDR15* UAS	TTTACTTATTATTTTCAGGGTCGCT
F2717	*PDR15* UAS	ACCATCGTGCCATACCTTAAC
F2718	*PDR15* TATA	GTCTTGTTGTAGATACGTCATATGC
F2719	*PDR15* TATA	GATAATTATAAGTGTGTTTGCTTGTGT
F2720	*PDR15* ORF	CTAACAGTGATCAATTCTCCAGTACGG
F2721	*PDR15* ORF	GAGGCGCTTAGATTCTTCCATACAC
F2722	*PDR16* Upstream	AGCAGAGAAGAAAGATGTTACGCAGCC
F2723	*PDR16* Upstream	TCGAGAACCTGCAGCGTTTGTCT
F2724	*PDR16* UAS	ATCGCTTATAAGCAGGGA
F2725	*PDR16* TATA TSS	GCAATAGGTTCATGCACTATATG
F2726	*PDR16* TATA TSS	TTCTAACGAAGAAGTGGTATTAGTCTA
F2727	*SNQ2* Upstream	CCTTGTTCCTAGTGGCGCTAATGG
F2728	*SNQ2* Upstream	GCAGGCTTCGAAACTATAATTGCGTC
F2729	*SNQ2* UAS Left	AGTGGCTCCGCACACAGAGGTTAT
F2730	*SNQ2* UAS Left	CGCGGATGCTAGATTTCTACGAGTCATA
F2731	*SNQ2* TATA	GTAGTGCCCCTCCCTCACCGCTAT
F2732	*SNQ2* TATA	TTTGACCTCGCAACACTTCAACTTGAAA
F2733	*YOR1* TATA	CTATACAAGAACAGGAAGAACGCATTTC
F2734	*YOR1* TATA	GACTTTTGTTTTCCAGCTCCGTC
F2735	*YOR1* Upstream	AATAATCTTCTGCTTTCTCTGCGTGTC
F2736	*YOR1* Upstream	CTTCGCATATCAAAGTGTCAATTAAGGA
F2996	*YOR1* UAS Left	AAAATCTATATGACTAACCGCAAGGG
F2997	*YOR1* UAS Left	AAACAGTAACTGCGTGCTATAAGGTG
F3159	*PDR15* Upstream	AGTACCGTGAAGAAAGACTTAACTGC

F3160	*PDR15* Upstream	GACGCTCTCGCTTATTGTCTATCT
F3161	*PDR15* UAS	TATTTTTGTTCGCGCAAAGAGCAAGA
F3162	*PDR15* UAS	GACTCTCCGCGGAACGAGCAT
F3189	*YOR1* ORF	AAGCGACAGAAGAAGAGGTTATGGA
F3190	*YOR1* ORF	GGGTTAAAACCGGATGTACAATTAGC
F3191	*PDR16* TATA	CATTCCACCCGACTCTATTTGAGTG
F3192	*PDR16* TATA	CAATATTGTGCATGTACAAGAGTGGGAT
F3198	*PDR5* ORF	CGTCAGAGGTGTTTCCGGTGGT
F3199	*PDR5* ORF	GGCACGAATAAATTCCAAAGCGGTAG
F3229	*PDR5* UAS R	AGCACCCTTTGGACTCGTGATTC
F3230	*PDR5* UAS R	AACAGATATCGTACCACGGCGTAGAA
RT-qPCR oligos		
F2173	*RDN25-1*	CGTTCCTTGTCTATGTTCCTTG
F2174	*RDN25-1*	CACTGTACTTGTTCGCTATCG
F2430	*RPR1*	CACCTATGGGCGGGTTATCAG
F2431	*RPR1*	CCTAGGCCGAACTCCGTGA
F2462	*PDR5*	TACCGCTTTGGAATTTATTCG
F2463	*PDR5*	GCATATCTTTGTTCAGGGTTC
F2466	*PDR16*	GTCGAGAACGAATCAGGTAAG
F2467	*PDR16*	GGTACTTTAGGAACATCAGGG
F2470	*SNQ2*	GTATCTGGTGGTGAACGTAAG
F2471	*SNQ2*	CTTGGAGGACACAAATAACCC
F2474	*YOR1*	TTCCTAACGGTAAAGTGACTTC
F2475	*YOR1*	GATGTTCGCAGCAATTCTAAA
F2720	*PDR15*	CTAACAGTGATCAATTCTCCAGTACGG
F2721	*PDR15*	GAGGCGCTTAGATTCTTCCATACAC
